# Steroid Metabolites Support Evidence of Autism as a Spectrum

**DOI:** 10.3390/bs9050052

**Published:** 2019-05-09

**Authors:** Benedikt Andreas Gasser, Johann Kurz, Bernhard Dick, Markus Georg Mohaupt

**Affiliations:** 1 Department of Clinical Research, University of Bern, 3010 Berne, Switzerland; bernhard.dick@gmx.ch (B.D.); markus.mohaupt@lindenhofgruppe.ch (M.G.M.); 2 Intersci Research Association, Karl Morre Gasse 10, 8430 Leibnitz, Austria; john@a1.net; 3 Division of Nephrology/Hypertension, University of Bern, 3010 Berne, Switzerland; 4 Teaching Hospital Internal Medicine, Lindenhofgruppe, 3006 Berne, Switzerland

**Keywords:** ACTH, autistic disorder, antiglucocorticoid, Kanner, Asperger

## Abstract

*Objectives*: It is common nowadays to refer to autism as a spectrum. Increased evidence of the involvement of steroid metabolites has been shown by the presence of stronger alterations in Kanner’s syndrome compared with Asperger syndrome. *Methods*: 24 h urine samples were collected from 20 boys with Asperger syndrome, 21 boys with Kanner’s syndrome, and identically sized control groups, each matched for age, weight, and height for comprehensive steroid hormone metabolite analysis via gas chromatography–mass spectrometry. *Results*: Higher levels of most steroid metabolites were detected in boys with Kanner’s syndrome and Asperger syndrome compared to their matched controls. These differences were more pronounced in affected individuals with Kanner’s syndrome versus Asperger syndrome. Furthermore, a specific and unique pattern of alteration of androsterone, etiocholanolone, progesterone, tetrahydrocortisone, and tetrahydrocortisol was identified in boys with Kanner’s syndrome and Asperger syndrome. Interestingly, in both matched samples, only androsterone, etiocholanolone, progesterone, tetrahydrocortisone, tetrahydrocortisol, and 5a-tetrahydrocortisol groups were positively correlated. In the Asperger syndrome group, all metabolites showed a positive correlation. In the Kanner’s syndrome group, 5-a tetrahydrocortisol with androsterone showed a positive correlation. *Conclusions*: Due to differences in the level of alteration, the premise that Asperger syndrome is on the mild side of the autism spectrum and that Kanner’s syndrome is on the severe side is supported, but alteration patterns yield different phenotypic expressions.

## 1. Introduction

Autism spectrum disorder (ASD) comprises a group of disorders characterised by persistent deficits in social communication and social interaction and restricted patterns of behaviour, interests and activities [1]. The aetiology of autism is not well understood, although it is thought to involve genetic, immunologic and environmental factors [1,2,3,4,5,6,7,8,9,10,11,12]. The diagnosis of autism is still based solely on behavioural characteristics as there is currently no biological marker [13]. In ICD-10 (International Classification of Diseases, 10th Revision) from the World Health Organisation (WHO), the premise of autism as a spectrum was adopted overcoming the previously widely used diagnoses of Kanner Syndrome respectively early-infantile autism (F84.0) and Asperger syndrome (F84.5).

Consensus was reached that autism was regarded as a spectrum with the former diagnosis of Asperger syndrome being a mild entity and Kanner syndrome a severe one. These former diagnoses are nowadays given less attention, however still lots of affected individuals having these diagnoses. Clinical evidence supports the premise of Asperger’s disorder as a mild form of autism sharing a common aetiology and developmental neuropathology with autism [14]. Involvement of epigenetic mechanisms, linking genes with the environment, was suggested by studies conducted on lymphoblasts from siblings with non-conforming autism diagnoses. They showed increased expression of genes associated with steroid biosynthesis in brain development [15]. From a more comprehensive standpoint, some studies suggest an involvement of the corticotropin-releasing hormone (CRH)-adrenocorticotropic hormone (ACTH) system with additional alterations of adrenal gland metabolites in autistic disorders [16,17,18,19,20,21].

The regulation of proangiogenic factors was investigated in adrenocortical cells isolated from human foetal adrenal glands for a more comprehensive understanding [22,23,24]. ACTH up-regulates vascular-endothelial growth factor–A (VEGF-A) and increases mRNA encoding of angiopoietin 1 (Ang1) whereby indicating that ACTH is the primary regulator of adrenal organ growth by stimulating VEGF and thus angiogenesis allowing to link the hypothalamic-pituitary adrenal axis (HPA-axis) with adrenal gland development [22,23,24].

To broaden our understanding it should be mentioned that higher rates of clinical manifestations of androgen-related conditions (e.g., polycystic ovary syndrome, hirsutism, acne and hormone-related cancers) were more often reported in populations with autism spectrum conditions (as clinical correlates of hyperandrogenism) compared to healthy non-autistic populations [25]. In particular, the relevance of testosterone for developmental disorders was shown for children from hyperandrogenic mothers with polycystic ovary syndrome (PCOS), increasing the risk of pervasive development disorders [26]. Especially prenatal androgen exposure was suggested to influence mental health leading to studies on psychiatric morbidity among individuals with congenital adrenal hyperplasia (CAH, the inherited inability to synthesie cortisol) [27]. Interestingly, individuals with 21-hydroxylase deficiency have an increased risk of psychiatric disorders with the highest risk among those with the most severe genotype and co-occurrence of CAH and autistic disorders [28]. Further evidence can be derived from animal models where it was shown that maternal hyperandrogenism during pregnancy increased the susceptibility of the foetus to autism spectrum disorder (ASD). In particular, the intrauterine environment played an important role in the development of ASD [29]. Despite these facts, drugs directly affecting the adrenal gland were suggested to improve clinical symptoms. Bradstreet et al. (2006) used Spironolactone as a desirable immunologic and hormonal intervention in ASD [30]. Recent work looking at the development of autism during gestation and childhood suggested that the placenta is capable of synthesizing sex steroids de novo from maternal substrates (cholesterol), maternal or foetal prohormones and steroid precursors [31]. Active steroids produced in the placental unit can be released into both the maternal and foetal compartments to maintain pregnancy and allow foetal development. In addition, endocrine disrupting compounds may be another source of environmentally mediated influences on the early foetal hormonal milieu and a predisposition for the development of autism [32]. Xu et al. (2015) studied the link between pre-term concentrations of adrenal hormones and behaviour. The authors found that female rats treated with androgens spent less time in social interaction in adolescence and exhibited impaired heterosexual interaction in adulthood. Furthermore, the duration of social and heterosexual interaction of female offspring was negatively correlated to maternal serum testosterone levels during pregnancy [29]. In summary, there seems to be clear evidence for a link between increased gonadal adrenal sex steroids (particularly testosterone, estradiol, dehydroepiandrosterone sulfate and androstenedione) in individuals with autism and autism-relevant behaviour, which implies that greater postnatal testosterone levels in early infancy are predictive of more male-typical behaviour later in life [25,33,34,35] Previous work on metabolite levels clearly showed the effects of testosterone on phenotypic variability related to autism. Foetal testosterone influenced individual differences in the typical development of eye contact, size of vocabulary, restriction of interests, mentalizing, empathy, attention to detail, urge to systemise, and autistic traits [36].

In summary, broad evidence exists for an involvement of adrenal gland metabolites in ASD, whereby patterns of severity might vary in individuals with Asperger compared to Kanner syndrome. This leads us to the aim of this study, which was to analyse the degree of involvement of adrenal gland metabolites and adrenal gland enzyme activities (21-hydroxylase, 17-hydroxylase and 11 beta-hydroxylase) in children with Asperger and Kanner syndrome. We hypothesised that adrenal gland metabolites and enzyme activities were altered to a larger extent in children with Kanner syndrome compared to Asperger syndrome [37].

## 2. Materials and Methods

### 2.1. Participants

Twenty boys diagnosed with Asperger syndrome (BMI 18.2 ± 3.2; average age 15.3 ± 2.9 years) with an individually pairwise matched control group (BMI 18.2 ± 3.5; average age 14.2 ± 2.4 years) and 21 boys diagnosed with Kanner’s syndrome (BMI 20.4 ± 5.9; average age 13.6 ± 3.6 years) with an individually pairwise matched control group (BMI 19.4 ± 3.1; average age 13.3 ± 3.1 years) were recruited.

### 2.2. Study Design

All procedures performed in studies involving human participants were in accordance with the ethical standards of the institutional and/or national research committee and with the 1964 Helsinki declaration and its later amendments or with comparable ethical standards. The study was approved by the governmental ethics board of Graz, Austria, and registered at ClinicalTrials.gov. Involvement in the study was voluntary and not compensated. After study procedures had been fully explained, the parents of the participants read and signed informed consent forms. Autistic and control boys were recruited from the area of Leipzig (Austria). Enrolment took place from mid-2009 to mid-2012. All participants were Caucasians. Participants were excluded if they had a neurological and psychiatric disorder other than autism and comorbid disorders, a history of liver diseases, renal or endocrine disorders, a current infection, or fever. Mental retardation or behavioural disorders were exclusion criteria only for the control group but were allowed as comorbid conditions in the autistic group. The diagnosis was given in the first years of life of the children according to the diagnostic criteria of the DSM-IV and was cross-checked by experienced clinicians (medical doctors and/or psychologists) during enrolment of the study. Key criteria such as the extent of language impairment were used in order to distinguish between Kanner’s syndrome and Asperger syndrome [38](A further tool used to increase the accuracy of diagnosis was the Marburg questionnaire for Asperger syndrome (MBAS).

### 2.3. Methods

Analysis of urinary steroids was conducted via gas chromatography–mass spectrometry. Urine samples were taken between 7 a.m. and 9 a.m. in the morning after breakfast. Urine sample preparation comprised pre-extraction, enzymatic hydrolysis, extraction from the hydrolysis mixture, derivatization, and gel filtration [39,40,41]. The recovery standard was prepared by adding 2.5 µg of medroxyprogesterone to 1.5 mL of urine. The sample was extracted on a Sep-Pak C18 column (Waters Corp., Milford, MA, USA), dried, reconstituted in a 0.1 M acetate buffer, pH 4.6, and hydrolysed with a powdered Helix pomatia enzyme (12.5 mg; Sigma Chemical Co., St. Louis, MI, USA) and 12.5 µL of β-glucuronidase/arylsulfatase liquid enzyme (Roche Diagnostics, Rotkreuz, Switzerland). The resulting free steroids were extracted on a Sep-Pak C18 cartridge. A mixture of internal standards (2.5 μg each of 5α-androstane-3α, 17α-diol, stigmasterol, and cholesterol butyrate, and 0.15 μg of 3β5β-tetrahydroaldosterone) was added to this extract, and the sample was derivatised to form the methyloxime-trimethylsilyl ethers. Analyses were performed on a Hewlett-Packard gas chromatograph 6890 (Hewlett Packard, Palo Alto, CA, USA) with a mass selective detector 5973 by selective ion monitoring (SIM). One characteristic ion was chosen for each compound measured. The derivatised samples were analysed during a temperature-programmed run (210–265 °C) over a 35 min period. The calibration standard consisted of a steroid mixture containing known quantities of all steroid metabolites to be measured. Responses and retention times were recorded regularly. In each case, the ion peak was quantified against the internal stigmasterol standard. With this method, the most relevant metabolites of the glucocorticoid synthesis pathway were measured and presented. All results were adjusted for creatinine in urine to check renal function.

### 2.4. Statistical Analysis

SPSS Version 21 and Microsoft Excel were used to analyse all data. A Student’s *t*-test was performed to compare the means of data from two groups. Results where *p* < 0.05 were considered significant. Data were analysed for normal distribution with a Jarque–Bera test, whereby the hypothesis of a normal distribution, at *p* < 0.1 for all samples, could not be rejected. Correlations between different metabolites and between classes of metabolites were calculated. Enzyme ratios and demographic data are presented as standard error of mean ± standard deviation. Clinical data are presented as mean ± SEM. Calculations were made with GraphPad Prism (GraphPad Software, Inc., La Jolla, CA, USA) and Microsoft Excel (Microsoft Inc., Redmond, WA, USA).

## 3. Results

**Figure 1:**
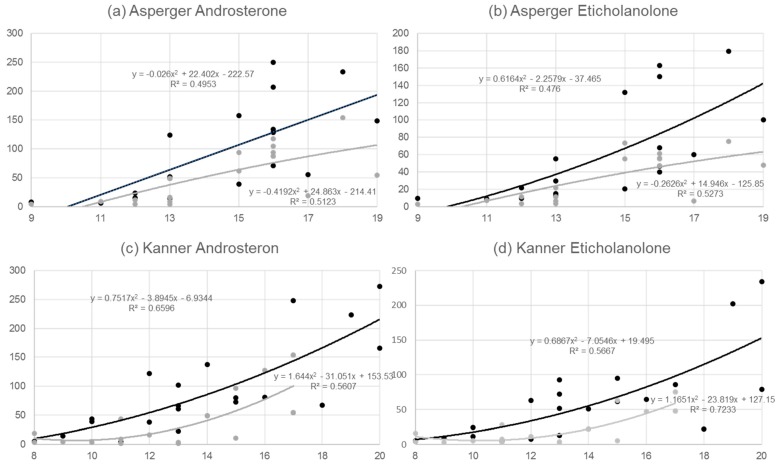
(**a**) Correlative relationship between age and androsterone (ratio of androsterone (mmol/L) to urine creatinine (µmol/L)) for *n* = 20 boys with Asperger syndrome (black line) and for *n* = 20 pairwise matched controls (grey line). (**b**) Correlative relationship between age and etiocholanolone (ratio of eticholanolone (mmol/L) to urine creatinine (µmol/L)) for *n* = 20 boys with Asperger syndrome (black line) and for *n* = 20 pairwise matched controls (grey line). (**c**) Correlative relationship between age and androsterone (ratio of androsterone (mmol/L) to urine creatinine (µmol/L)) for *n* = 21 boys with Kanner syndrome (black line) and *n* = 21 pairwise matched controls (grey line). (**d**) Correlative relationship between age and etiocholanolone (ratio of androsterone (mmol/L) to urine creatinine (µmol/L)) for *n* = 21 boys with Kanner syndrome (black line) and *n* = 21 pairwise matched controls (grey line).

Metabolite concentrations of adrenal gland hormones differed in boys with Asperger syndrome and Kanner’s syndrome compared to their matched control groups regarding androgens, 11-deoxycortisol, corticosterone metabolites, and cortisone metabolites as well as oestrogens and progesterones (Table 1). Enzyme activities were also different in boys with Asperger syndrome and those with Kanner’s syndrome compared to their matched controls (Table 2). These differences were more pronounced in the Kanner’s syndrome group. Bivariate correlation matrices were calculated for the most important hormones, i.e., those that exhibited highly significant differences in order to identify varying patterns in Asperger syndrome and Kanner’s syndrome (Table 3). Furthermore, correlations between the whole class of metabolites (androgens and cortisol metabolites) were determined. A general dysregulation of androgens is apparent: correlation analysis of all androgen metabolites showed a correlative relationship of 0.959 in individuals with Asperger syndrome versus the controls and a correlative relationship of 0.911 in individuals with Kanner’s syndrome versus the controls (Asperger syndrome and Kanner’s syndrome correlated with 0.943, and the two control groups correlated with 0.973). When analyzing all cortisol metabolites, markedly higher correlative relationships between the controls and Asperger syndrome (0.987) and between the controls and Kanner’s syndrome (0.997) were identified, further supporting the dysregulation of androgen metabolites. Special attention was paid to androsterone and etiocholanolone, the two highly increased androgens (Figure 1a–d).

## 4. Discussion

This study investigated alterations in adrenal gland metabolites and adrenal gland enzyme activities in boys with Asperger syndrome (F84.5) and Kanner’s syndrome (early-infantile autism-F84.0) [42,43]. Although a relatively small cohort of affected individuals was analysed, limiting the general validity of the results, consistent patterns can be detected. All classes of metabolites showed differences between children with autism (Asperger syndrome and Kanner’s syndrome) and their individually pairwise matched control groups, supporting the hypothesis of a principal involvement of adrenal gland tissue in autism spectrum disorder. In particular, androgens, 11-deoxycortisone, corticosterone, cortisol, cortisone, oestrogen, and progesterone metabolites varied significantly.

In principle, there is some evidence that Asperger syndrome and Kanner’s syndrome are distinguishable entities [38,44]. For example, it was revealed that adrenotropic metabolites such as epinephrine and norepinephrine but also chemical relatives such as tyrosine and homovanillic acid showed different patterns in individuals with Kanner’s syndrome compared to Asperger syndrome, clearly indicating differences in the severity of alterations [44]. In the present study, significant differences between individuals with autism and controls were more obvious in the case of Kanner’s syndrome compared to Asperger syndrome (e.g., 11B-hydroxyandrosterone and 5-a testosterone). Furthermore, for 11B-hydroxyeticholanolone, 5-androstene-3β, 17β-diol, and dehydroepiandrosterone, there were significant differences compared to matched controls in relation to Kanner’s syndrome but not to Asperger syndrome. This is also supported by the correlation analysis of all metabolites of a conducted class, indicating a stronger deviance from normal androgen concentrations in relation to Kanner’s syndrome compared to the Asperger syndrome, which implies that Kanner’s syndrome is a more severe form of autism.

The analysis of the two androgens, androsterone and etiocholanolone, showed around 50% increased values in boys with Asperger syndrome as well as Kanner’s syndrome compared to control groups (*p* = 0.06 for Asperger syndrome and *p* = 0.14 for Kanner’s syndrome) (Table 1). These two metabolites depend highly on testosterone. Testosterone is converted to androstenedione first and then to androsterone (5α-androstan-3α-ol-17-one; 5α,3α-A) and etiocholanolone (5β-androstan-3α-ol-17-one; 5β,3α-A) [45] (5α-Reductase (5α-R) and 5β-reductase (5β-R) catalyse the rate-limiting irreversible initial steps, which are followed by sequential reductions by 3α-hydroxysteroid dehydrogenase (3α-HSD) and 17β-hydroxysteroid dehydrogenase (17β-HSD) [45].

Although analyses in Figure 1a–d methodologically pursue a linear approach, this stands somehow in contrast to evidence of the strong increase of androgens in boys when puberty starts [46], which we were able to confirm with additional polynomial analysis. It is obvious from Figure 1a–d that the slope in the Asperger syndrome group was clearly higher than in the matched controls, whereas a parallel development over time was detected in the Kanner’s syndrome group and their controls. This implies differences in metabolic alterations over time in the two ASDs.

Androsterone and etiocholanolone showed the highest correlation in all metabolite groups in the bivariate correlation matrices (Table 3). Both control groups had a very similar pattern, with negative correlations of progesterone, androsterone, and etiocholanolone versus tetrahydrocortisol, 5-a tetrahydrocortisol, and tetrahydrocortisone. Interestingly, all metabolites in the Asperger syndrome group showed positive correlations, whereas in the Kanner’s syndrome group the pattern was similar to that of the matched controls. The absent negative correlative relationships in Asperger syndrome for the precursor progesterone and the androgens androsterone and etiocholanolone and the glucocorticoid groups of tetrahydrocortisol, 5-a tetrahydrocortisol, and tetrahydrocortisone stand out, suggesting a general dysregulation of steroid pathways. Interestingly, values for androsterone and etiocholanolone were higher in the Asperger syndrome group than in the Kanner’s syndrome group, whereas the opposite was observed for tetrahydrocortisone, tetrahydrocortisol, 5α-tetrahydrocortisol, and alpha-cortol.

Interestingly, in the 1970s there were some thoughts that the ratios of androsterone and etiocholanolone discriminated between heterosexual and exclusively homosexual individuals, clearly indicating that androsterone and etiocholanolone lead to a new sexual phenotype [47]. Looking at enzyme activities, especially 11-beta dehydrogenase (11-beta HSD), there was a striking difference in the ratio of (THF + 5aTHF)/THE between both Asperger syndrome (21.5) and Kanner’s syndrome (19.7) groups and their matched controls (2.8 and 1.3, respectively) (Table 2). Values larger than 3 were regarded as pathologic [41]. The ratio of (F + E)/(THE + THF + 5aTHF) measured in the present study indicated an alteration in beta-dehydrogenase activity. This ratio was about 15 times lower in the Asperger syndrome group and 20 times lower in the Kanner’s syndrome group compared to their matched controls, implying that not only adrenal gland metabolites but also enzyme activities were affected.

When aiming for an aetiopathogenetic understanding of the dysregulation of adrenal gland metabolites pre-term birth might provide an answer. From a morphometric point of view, pre-term birth might be a risk factor for adrenal gland dysregulation, as some studies found that during antenatal and neonatal periods the adrenal gland might shrink more rapidly in infants born at full term [48]. Interesting similarities can be observed when comparing our results to those of a recent study on levels of Δ4 steroids from amniotic fluid samples. The concentration of Δ4 sex steroids (progesterone, 17α-hydroxy-progesterone, androstenedione, and testosterone) and cortisol were positively associated with autism (Baron-Cohen et al. 2015). Furthermore, the principal component analysis confirmed that one generalised latent steroidogenic factor was driving much of the variation in the data. The autism group showed elevations across all hormones on this latent generalised steroidogenic factor, and this elevation was uniform across the ICD-10 diagnostic label [34]. This work was consistent with prior findings of interactions between the hypothalamic-pituitary adrenal axis (HPA-axis) and its known foetal programming effects on later atypical neurodevelopmental phenotypes [49]. There are probably more than 30 steps involved in adrenal gland metabolomics, from the first cyclised sterol lanosterol to the most downstream product estradiol [40]. Our analyses mainly focused on the metabolic steps for significantly different metabolites. The results presented here clearly showed an involvement of 17-hydroxylase, 21-hydroxylase, and 11β-hydroxylase in boys with Asperger syndrome as well as those with Kanner’s syndrome, indicating a principal involvement of adrenal gland tissue in ASD. In summary, a number of hormones and enzymes were altered in our samples from the Asperger syndrome group and the Kanner’s syndrome group rather than the abnormality being restricted to a specific steroid hormone. This observation suggests a dysregulation of pathways mediated by cytochrome P450-containing enzymes that catalyse the conversion of hormones along the glucocorticoid pathways [40]. Evidence pointing toward the importance of such enzymes was previously found via genetic associations between autism and single-nucleotide polymorphisms in CYP17A1, CYP19A1, and CYP11B1 genes [50]. The results of our sample of autistic children can be well embedded in these findings, suggesting an involvement of P450 oxidoreductase deficiency and an alteration in cortisone reductase activity.

When broadening the analysis to principal growth processes in childhood, it can be mentioned that, for children with autism, significantly greater head circumferences and greater weights than those in the control groups were found [51]. Furthermore, levels of insulin-like growth factor 1 (IGF-1), insulin-like growth factor 2 (IGF-2), insulin-like growth factor binding protein (IGFBP-3) and Growth Hormone (GH) binding protein which are all affected by cortisol, were significantly higher in autistic children than in controls supporting the point of view of an involvement of CRH-ACTH adrenal axis [51]. In conclusion, our data clearly showed altered adrenal gland metabolites in children with Asperger syndrome as well as Kanner’s syndrome compared to matched control groups, suggesting a principal involvement of adrenal gland tissue in ASD. Further evidence can be derived from the ratios of measured metabolites, which clearly indicate a dysregulation of enzyme activities. Clear differences between the Asperger syndrome group and the Kanner’s syndrome group emerged for the calculated ratios of androsterone and etiocholanolone, which was further supported by the calculated correlations of hormones with highly significant differences.

To sum up, results suggest different patterns in the alteration of adrenal gland metabolites in relation to Asperger syndrome and Kanner’s syndrome. Steroid hormone profiles, especially those of androgens, might be useful clinical markers that support the diagnoses of autism. The results further imply that mild and more severe forms of autism can be distinguished. For severe forms, steroid metabolite pathways might be a promising target for pharmaceuticals such as statins and may inhibit the cholesterol precursor of steroid pathways.

## Figures and Tables

**Table 1:** Androgen metabolites for boys with Asperger syndrome (*n* = 20) versus individually pairwise matched controls (*n* = 20) and boys with Kanner syndrome (*n* = 21) versus individually pairwise matched controls (*n* = 21).

	Asperger		*p*-Value			Kanner		*p*-Value		
	Boys			Controls		Boys			Controls	
	Mean	SEM		Mean	SEM	Mean	SEM		Mean	SEM
**Androgen Metabolites**										
Androsterone	95.43	18.36	0.008	57.7	9.79	89.29	17.4	0.001	61.45	13.67
Etiocholanolone	62.45	13.21	0.043	38.92	6.16	59.78	13.45	0.009	29.23	6.24
Androstenediol	3.35	0.66	0.056	2.2	0.41	3.58	0.7	0.087	2.88	0.6
11-Oxoetiocholanolone	26.53	3.48	0.667	24.31	2.86	30.93	6.96	0.119	19.45	2.05
11β-Hydroxyandrosterone	32.84	3.57	0.07	26.2	3.15	46.78	7.57	0.033	30.72	4.41
11β-Hydroxyetiocholanolone	18.76	3.15	0.913	19.3	2.78	23.1	4.57	0,08	14.5	1.91
Dehydroepiandrosterone	6.02	2.65	0.72	4.92	1.31	36.82	19.5	0.129	7.57	2.03
5-Androstene-3β,17β-diol	3.67	0.57	0.538	3.19	0.55	7.13	2.67	0.191	3.85	0.73
16α-Hydroxydehydroepiandrosterone	11.05	3.31	0.311	20.16	7.29	16.32	4.42	0.506	21.26	6.88
5-Androstene-3β,16α,17β-triol	18.73	4.53	0.537	23.24	5.44	19.31	4.06	0.504	23.23	5.18
5-Pregnene-3β, 16α,17β-triol	6.41	2.21	0.739	7.21	1.22	10.55	5.2	0.696	8.66	1.33
Testosterone	2.93	0.71	0.056	1.46	0.32	3.01	1	0.134	1.62	0.34
5α-Dihydrotestosterone	1.29	0.17	0.051	0.96	0.12	1.66	0.24	0.035	1.16	0.15
**Oestrogen metabolites**										
Estriol	0.14	0.02	0.859	0.14	0.03	0.18	0.04	0.288	0.16	0.02
17β-Estradiol	0.1	0.02	0.043	0.05	0.01	0.09	0.02	0.505	0.08	0.01
**Progesterone metabolites**										
17-Hydroxypregnanolone	5.43	0.86	0.068	3.32	0.76	7.34	1.32	0.005	3.7	0.46
Pregnanediol	10.31	0.95	0.37	8.91	2.05	13.45	1.61	0.019	9.41	1.16
Pregnanetriol	32.62	4.95	0.043	22.17	5.1	35.15	5.09	0.001	16.48	1.98
11-Oxopregnanetriol	1.41	0.29	0.482	1.1	0.25	2.01	0.57	0.108	0.99	0.16
**11-Deoxycortisol Metabolite**										
Tetrahydrodeoxycortisol	0.48	0.04	0.588	0.44	0.07	0.68	0.09	0.031	0.47	0.03
**Corticosterone metabolites**										
Tetrahydrodihydrocorticosterone	12.83	1.42	0.093	9.38	1.26	14.72	1.86	0.014	9.64	0.63
Tetrahydrocorticosterone	11.77	1.22	0.193	9.57	1.14	15.6	1.57	0.005	10.22	0.85
5α-Tetrahydrocorticosterone	26.8	3.73	0.096	19.67	2.13	47.71	8.2	0.015	24.66	2.2
18-Hydroxytetrahydrodihydrocorticosterone	4.53	0.51	0.364	3.34	0.58	6.24	1	0.073	3.28	0.67
Tetrahydroaldosterone	2.38	0.33	0.002	1.24	0.1	1.96	0.23	0.096	1.48	0.15
**Cortisone Metabolites**										
Cortisone	10.8	1.34	0.592	11.86	1.18	13.74	1.91	0.884	13.42	1.46
Tetrahydrocortisone	245.62	21.04	0.005	171.72	12.04	278.77	27.31	0.007	185.14	16.29
α-Cortolone	84.51	6.91	0.002	59.54	3.65	99.76	16.05	0.031	63.1	4.78
β-Cortolone	0.69	0.07	0.001	110.39	18.11	0.73	0.1	0	137.5	25.01
20α-Dihydrocortisone	1.3	0.19	0.989	1.29	0.13	1.64	0.28	0.784	1.55	0.19
20β-Dihydrocortisone	3.79	0.49	0.849	3.92	0.39	4.88	0.79	0.932	4.96	0.61
**Cortisol metabolites**										
Cortisol	8.72	1.4	0.209	6.33	0.65	12.72	2.22	0.044	7.36	1.02
Tetrahydrocortisol	76.84	6.13	0.016	56	4.02	106.23	9.53	0.001	63.06	6.38
5α-Tetrahydrocortisol	102.92	14.16	0.017	60.91	4.07	146	20.78	0.005	79.77	6.97
α-Cortol	16.02	1.23	0.002	10.82	0.69	22.09	2.31	0.001	12.34	0.9
β-Cortol	21.11	1.61	0.125	16.93	2.11	29.17	2.97	0.009	17.18	2.35
20α-Dihydrocortisol	3.28	0.31	0.556	3.71	0.55	5.91	1.15	0.297	4.55	0.7
6β-Hydroxycortisol	9.68	0.85	0.754	9.13	1.39	19.58	6.07	0.095	9.51	1.47
18-Hydroxycortisol	26.34	1.95	0.54	23.3	124	53.56	15.9	0.245	32.7	6.02

**Table 2:** Enzyme activities for boys with Asperger syndrome (*n* = 20) versus individually pairwise matched controls (*n* = 20) and boys with Kanner syndrome (*n* = 21) versus individually pairwise matched controls (*n* = 21). Data given as ratio of metabolites (mmol/L) to urine creatinine (µmol/L). (THA = Tetrahydrodehydrocorticosteron; THB = Tetrahydrocorticosteron; 5aTHB = 5a-Tetrahydrocorticosteron; THS = Tetrahydrosubstance S; HP = 17-Hydroxypregnalonon; PT = Pregnanetriol; PT’ONE = 11-Oxo-Pregnanetriol; THDOC = Tetrahydrodoc; E = Cortison; THE = Tetrahydrocortison; F = Cortisol; THF = Tetrahydrocortisol; 5ATHF = 5-alphaTetrahydrocortisol).

	Asperger Boys		*p*<	Control Boys		Kanner Boys		*p*<	Control Boys	
	Mean	SD		Mean	SD	Mean	SD		Mean	SD
**21-Hydroxylase Deficit**										
17HP/(THE + THF + 5aTHF)	0.014	0.01	0.684	0.013	0.01	0.004	0.021	0.711	0.014	0.02
PT/(THE + THF + 5aTHF)	0.009	0.007	0.17	0.007	0.005	0.016	0.108	0.00	0.005	0.003
100 × PT’ONE/(THE + THF + 5aTHF)	0.003	0.004	0.000	0.357	0.377	0.002	0.001	0.00	0.532	0.481
**17-Hydroxylase Deficit**										
(THA + THB + 5aTHB)/(THE + THF + 5aTHF)	0.121	0.035	0.000	0.238	0.098	0.085	0.159	0.00	0.259	0.085
100 × THS/(THE + THF + 5aTHF)	0.016	0.006	0.000	1.00	0.673	0.012	0.018	0.00	1.08	0.478
**11-Hydroxylase Deficit**										
100 × THDOC/(THE + THF + 5aTHF)	0.001	0.000	0.000	7.147	5.212	0.042	0.158	0.00	7.241	3.54
**11-Beta HSD Deficit**										
F/E	0.786	0.203	0.000	0.386	0.197	0.175	0.793	0.009	0.339	0.088
(THF + 5aTHF)/THE	21.517	14.141	0.000	2.821	4.513	19.736	9.452	0.00	1.362	0.471
(F + E)/THE + THF + 5aTHF	0.048	0.028	0.000	1.57	0.658	0.035	0.079	0.00	1.797	0.563

**Table 3:** Correlation matrices depicting the relationship between the significantly different hormones in (**a**) Asperger *n* = 20 (**b**) Kanner *n* = 21 (**c**) Asperger controls *n* = 20 (**d**) Kanner controls *n* = 21. Abbreviations: Andro = Androsterone, Etio = Etiocholanolone, PT = Progesterone, THE = Tetrahydrocortisone, THF = Tetrahydrocortisol, 5aTHF = 5a-Tetrahydrocortisol. Dark green correlation between 0.5 < correlation < 1, light green 0 < correlation < 0.5, red correlation is negative.

	ANDRO	ETIO	PT	THE	THF	5ATHF
**(a) Asperger**						
**ANDRO**	1.00	0.81	0.81	0.14	0.36	0.32
**ETIO**	0.81	1.00	0.71	0.39	0.39	0.39
**PT**	0.81	0.71	1.00	0.39	0.40	0.39
**THE-**	0.14	0.39	0.39	1.00	0.90	0.39
**THF**	0.36	0.39	0.40	0.90	1.00	0.59
**5ATHF**	0.32	0.39	0.39	0.39	0.59	1.00
**(b) Kanner**						
**ANDRO**	1.00	0.98	0.96	−0.34	−0.43	0.20
**ETIO**	0.98	1.00	0.95	−0.33	−0.33	−0.33
**PT**	0.96	0.95	1.00	−0.42	−0.38	−0.41
**THE**	−0.34	−0.33	−0.42	1.00	0.91	0.64
**THF**	−0.43	−0.33	−0.38	0.91	1.00	0.72
**5ATHF**	0.20	−0.33	−0.41	0.64	0.72	1.00
**(c) Asp. Cont.**						
**ANDRO**	1.00	0.91	0.84	−0.32	−0.30	−0.11
**ETIO**	0.91	1.00	0.92	−0.23	−0.23	−0.23
**PT**	0.84	0.92	1.00	−0.12	−0.15	0.91
**THE-**	−0.32	−0.23	−0.12	1.00	0.91	0.79
**THF**	−0.30	−0.23	−0.15	0.91	1.00	0.72
**5ATHF**	−0.11	−0.23	0.91	0.79	0.72	1.00
**(d) Kan. Cont.**						
**ANDRO**	1.00	0.93	0.82	−0.27	−0.35	−0.03
**ETIO**	0.93	1.00	0.93	−0.33	−0.42	−0.15
**PT**	0.82	0.93	1.00	−0.22	−0.15	−0.19
**THE-**	−0.27	−0.33	−0.22	1.00	0.90	0.52
**THF**	−0.35	−0.42	−0.15	0.90	1.00	0.54
**5ATHF**	−0.03	−0.15	−0.19	0.52	0.54	1.00
